# Maternal health-seeking behaviour of peri-urban women living with disability in Busiro South Health sub District, Wakiso district, Uganda: a community-based study

**DOI:** 10.4314/ahs.v22i4.45

**Published:** 2022-12

**Authors:** Bonny Natukunda, David Musoke, Arthur Kiconco, Samuel Mugambe, Christine Atuhairwe, Ivan Mugisha Taremwa, Miisa Nanyingi

**Affiliations:** 1 Faculty of Health Sciences, Uganda Martyrs University, Nkozi, Uganda; 2 Department of Disease Control and Environmental Health, School of Public Health, Makerere University College of Health Sciences, Kampala, Uganda; 3 Institute of Public Health and Management, Clarke International University, Kampala, Uganda; 4 Institute of Allied Health Sciences, Clarke International University, Kampala, Uganda

**Keywords:** Peri-urban, maternal health-seeking behaviour, disabled women, Uganda

## Abstract

**Aim:**

We examined the maternal healthcare-seeking behaviour of peri-urban women with disabilities in Busiro South Health Sub District, Wakiso district, Uganda.

**Methods:**

This community-based cross-sectional study. Data were collected using semi-structured questionnaires and focus group discussions. Chi-square was used to test for differences, and logistic regression to determine factors associated with maternal health-seeking behaviour at a 5% level of significance. Content analysis was used to analyse qualitative data.

**Results:**

A total of 182 women with disabilities were enrolled. Overall, 150 (82.3%) of the disabled women had attended ANC and 147 (80.8%) had delivered their babies at a health facility. The participants' mean age was 31.9±7.8years (range: 17–49 years). ANC attendance among disabled women was influenced by maternal age 18–30 years (p = 0.010), number of times a disabled woman was pregnant (p = 0.003), parity (p = 0.018), a normal delivery (p = 0.048), receiving financial support from friends of partners (p < 0.001), distance of less than 5KM to the health facility (p = 0.024), friendliness of the health care providers (p=0.030) and availability of health providers (p < 0.001).

**Conclusion:**

There is an urgent need for a multi-sectoral approach to better healthcare-seeking behaviour.

## Background

Women with a physical and visual disability during pregnancy remain at a heightened risk for pregnancy-related health complications^1^,^2^. Despite the SMDGs agenda to improve maternal health by saving the lives of more than half a million women who die as a result of complications during pregnancy through 2030, women with disabilities face challenges accessing healthcare and support before, during, and following their pregnancies, which adds to these health disparities^2^. In sub-Saharan countries like Uganda, the specific needs of women with disabilities may not be addressed in the mainstream pregnancy like providers do not have the needed equipment (like adjustable examination tables, accessible scales) to provide adequate reproductive care, pregnancy, and parenting information for women with disability (books, magazine, health education programs) and inadequately trained healthcare providers among others^2^.

Globally, many women with disabilities are becoming pregnant/mothers, and yet their care during pregnancy and beyond needs improvement^27^. Disabled women are just as likely to pregnant as those without disabilities, but it's unknown how these women navigate through pregnancy, childbirth, and motherhood^28^. For instance, women with disabilities face unique challenges because most clinicians do not have adequate information to manage their pregnancies & postpartum support, are misinformed about their disabilities, physical inaccessibility of health facilities (inaccessible offices & equipment like examining tables, hospital beds, weight scales), express negative harmful stereotypes about their disability and sexuality^28^. More so, disabled women due to isolation, have a high risk for poor birth outcomes like low birth weight and premature births among others^28^. However, these challenges can be minimized by the dissemination of information on pregnancy and childbirth, ensuring appropriate communication and understanding, and supporting women's sense of control to build trusting relationships with healthcare providers^27^.

In recent years, the pregnancy rates among women with disabilities have grown and are similar to the pregnancy rates of women without disabilities in the same age and income groups^3^. Evidence from five countries in 2014 showed that on average, births from mothers with disabilities were slightly less likely to be attended to by a skilled health worker from mothers without disabilities (71% versus 74%)^4^. The widest gap was found in Uganda-8 percent points where 66% of births from mothers with disabilities versus 74% from mothers without disabilities were attended to by skilled health workers^1^, ^4^. This may be attributed to the negative attitudes of skilled health workers and lack of awareness of mothers with disabilities, for vital information on such services may not be accessible formats^1^. In Africa, women with disabilities are more affected due to limited availability of appropriate health services especially in rural and remote areas, uneven access to buildings (hospitals, health centers, narrow doorways, inadequate bathroom facilities), inadequate skills and knowledge of health workers to meet their needs^4^. Consequently, this affects their maternal and reproductive health-seeking^5^, ^6^.

Although most women with disabilities attend antenatal care (ANC) during pregnancy, a bigger number deliver their babies in the hands of Traditional Birth Attendants (TBAs) due to the fear of being mistreated by health care providers and other women^6^, ^7^. The gap between births to mothers with and without disabilities could be due to income disparities and the subsequent greater inability of mothers with disabilities to afford this service^1^. Additionally, affordability of health services and transportation are the two main reasons why women with disabilities do not receive much maternal health in low-income countries^5^. The proportion of women with disabilities who give birth is on the rise despite the little attention given to them^8^. According to the UN disability report of 2019, skilled birth professionals attended to 64% of birth from mothers with disabilities in rural areas versus 83% living in urban areas. The gap was wide in Gambia (30%), where only 35% of the births were from mothers with disabilities in rural areas who were assisted by a skilled health worker during childbirth^1^.

Conversely, other studies show numerous challenges faced by women with disabilities that include unaffordable health facilities (58.7%), no transport means to the health facility (16%), and 25.8% could not afford prohibitive transport costs^9^. The distance to health care facilities is a barrier for mothers with disabilities in Africa because public transport is often inaccessible and unreliable, while private transportation is expensive^10^,^11^,^1^. Furthermore, the need for mothers with disabilities to be accompanied by a health visit increases transportation costs and raises issues of confidentiality for many^10^, ^11^.

In Uganda, 14.5% of persons with disabilities are women^12^, and those of reproductive age are more susceptible to poor pregnancy outcomes due to their anatomical nature 5. This may be a contributing factor to current Uganda's Maternal Mortality Ratio (MMR) of 336 per 100,000 live births^8^ that is minimizable by addressing physical barriers, especially hospital buildings, removal of prohibitive costs (transport costs and user fees), and equipping health workers to handle disability. In Uganda, efforts to better maternal outcomes are focused on improving access and quality of women's care without regard to the disabled^13^. Furthermore, poor understanding of the needs of disabled women, inadequate information, physical barriers, unfriendly health care providers, and suboptimal healthcare services have led to poor pregnancy outcomes among women with disabilities^14^. As there is limited information regarding the accessibility to maternal health care services by women living with disabilities, likely faces significant unmet needs^4^. In Wakiso district, anecdotal evidence seems to suggest that many of these women with disabilities do not attend ANC and delivery at health facilities due to stigma. Under ideal situations, one would expect all planning and provision of services (both general and specific) to take care of the needs of people with disabilities, but this is not the case. This study explored the maternal healthcare-seeking behavior of women with disabilities in Busiro Health Sub District (HSD), Wakiso district, Uganda.

## Methods

### Study design and data collection

We conducted a cross-sectional study that collected data using quantitative and qualitative methods. For the quantitative data, we employed a semi-structured questionnaire for women with disabilities in which we captured the data of those who had children. For the qualitative component, focused group discussions (FGDs) were held with relatives of disabled women and key informant interviews were done with midwives and in-charges of health facilities in Busiro HSD to enrich and triangulate the quantitative results. Busiro South HSD is one of the 7 HSDs (Makindye Ssabagabo, Entebbe municipality, Kyadondo North, Kyadondo East, Busiro East, Busiro North, and Busiro South) located in Wakiso district. It is predominantly peri-urban, comprised of 28 parishes and 141 villages with 6.6% disabled people^10^. Busiro South was selected for the study because it had the highest number of women with disabilities in the district according to the 2014 census^12^.

### Study population sampling and recruitment

The study population comprised disabled women aged 15–49 years who had given birth (2017–2018) residing in Busiro HSD. Disability (WHO) has three dimensions: impairment in a person's body structure or function, or mental functioning (e.g., loss of limbs and loss of vision); activity limitations, such as difficulty seeing, hearing, walking, or problem-solving; and participation restrictions in normal daily activities, such as walking, engaging in social and recreational activities or obtaining health care and preventive services. In this study, women with disabilities were those with body structure impairment and visual disability. This category of women was identified with the guidance of Community Health Workers (CHWs) residing in the selected villages. We excluded disabled women who had given birth more than 3 years ago because it would be erroneous to measure maternal health-seeking behaviour. In addition, disabled mothers that had poor cognitive abilities were excluded. We determined the sample size (n=186) using the Kish formula (1965). Four FGDs were held with disabled women selected from the community, and 6 health care providers that were directly involved in ANC and at delivery were purposely selected as key informants. These FGDs and key informant interviews were sufficient to achieve data saturation.

### Study variables

Our outcome variables were ANC attendance and place of delivery, measured on a dichotomous scale (YES or NO). A disabled woman was defined as physically handicapped (mostly limbs) and visually impaired (blind). We defined ANC as the proportion of disabled women that had attended ANC (ANC attendance was determined by the Uganda Ministry of Health (MOH guidelines, 2018) standard of eight times during pregnancy in comparison with those who visited facilities less time or not at all for the most recent pregnancy). The independent variables include maternal age, marital status, number of pregnancies, children ever born, gestation period, having a normal delivery, receiving support from family and friends, distance to the health facility, attitude, and availability of health providers.

### Data collection

#### Quantitative data

We used semi-structured questionnaires for all eligible participants who were identified with the aid of Community Health Workers (CHWs) residing in the selected villages.

#### Qualitative data

We held six FGDs (8 members each), each comprising of Community health workers (CHWs) relatives of women with a disability, these were purposefully selected. The FGDs were held within the villages in the local language, “Luganda” with the aid of two research assistants and BN, the principal researcher. The principal researcher moderated all FGDs while her assistant audio recorded the responses and probed where necessary. The FGDs lasted 1 hour on average. The moderator ensured that all disabled women gave their opinions on ANC and delivery services as much as possible, irrespective of the correctness. In addition, the researcher minimized individual dominance during the sessions by probing other members of the group that were not engaged in the discussions. Regarding key informants' interviews, six healthcare workers were recruited and comprised of four midwives and two hospitals in charge directly involved with ANC clinics and delivery unit. They were purposefully selected and interviewed to elicit their opinions of ANC provision and skilled birth attendance. The key informant interviews conducted in English lasted at least 30 minutes each and were held at the respective health facilities in Busiro HSD.

### Data management and analysis

#### Quantitative data

Quantitative data were entered and analysed using SPSS version 21 IBM statistical software. We analysed categorical data using frequencies and percentages. The outcome variables were computed as the proportion of disabled women that attended ANC and place of delivery under a skilled health worker.

We used the chi-square to test for differences in categorical data with an expected cell count above 5 and Fisher's exact test if this assumption was violated. Only variables with a probability value of less than 5% were considered for multivariate analysis. We then used logistic regression (odds ratios with subsequent 95% confidence intervals (CI)) to identify factors associated with the outcomes.

#### Qualitative data

We transcribed the audio recordings and ensured accuracy by correlating them with the transcribed information. Four reviewers BN (UMU MPH graduate student), MN (Research supervisor), DM (Public health specialist, Makerere), and CA (Social scientist) read the transcripts independently and guided the interpretation using content analysis. BN and MN discussed the categories in the groups and developed explicit summaries describing the details given by the disabled women. The verbatim quotations were then used to enrich the quantitative results of the study.

### Ethical considerations

We received administrative approval to conduct the study from Uganda Martyrs University and the Wakiso District Health Officer. Participation in the study was voluntary, and all those involved provided written informed consent after BN explained the purpose of the study. We ensured confidentiality and privacy through unique identifies during data collection.

## Results

### Background characteristics of mothers living with disability

A total of 182 women with disabilities were obtained in Busiro HSD with a response rate of 95.3%. Overall, 150 (82.3%) of the disabled women had attended ANC during pregnancy and 147 (80.8%) had delivered their babies at the health facility. The mean age of women living with disabilities was 31.9± 7.8years (range: 17–49years). Of the 182 women living with disabilities, 98(53.8%) were above 30 years. Slightly less than half, 47.8% were married. More than half, 101(55.5%) had between 1–3 pregnancies and 119(65.4%) had 1–3 children. Most (150, 82.4%) of the women living with a disability had attended ANC while half 77(51.3%) started ANC at less than 20 weeks of gestation. Almost half of the disabled women (43.8%) had no means of transport to visit the public health facility. There were 122 (67.0%) who had received support from friends or relatives to be able to access ANC services. More than half 107(58.8%) of women with a disability had delivered their babies under skilled birth attendants at health facilities and 151(83.0%) had normal deliveries. More than half 82(51.9%) received support from their families and 122(67.0%) got support from a friend. 107(58.8%) of the disabled women reside more than 5 kilometres from the health facility in Busiro HSD. Concerning health workers, 95(52.2%) reported that health workers were friendly, and more than half 103(56.6%) were available at the health facility (Table 1).

**Table 1:** Maternal service utilization among women with disabilities in Busiro HSD

	ANC attendance			Place of delivery			

Characteristics	Yes(n=150)	No(n=32)	p- value	Hospital	Others	All (n=182)	P-value
Maternal Age							
18–30 years	76(50.7)	8(25.0)	.008*	75(51.0)	9(25.7)	84(46.2)	.008*
Above 30 years	74(49.3)	24(75.0)		72(49.0)	26(74.3)	98(53.8)	
Marital status							
Single	28(18.7)	3(9.4)	.347	29(19.7)	2(5.7)	31(17.0)	.019*
Married	73(48.7)	14(43.8)		73(49.7)	14(40.0)	87(47.8)	
Separated	36(24.0)	12(37.5)		32(21.8)	16(45.6)	48(26.4)	
Widowed	13(8.7)	3(9.4)		13(8.8)	3(8.6)	16(8.8)	
Number of pregnancies							
1–3	91(60.7)	10(31.3)	.002*	88(59.9)	13(37.1)	101	.022f*
4 or more	59(39.3)	22(68.8)		59(40.1)	22(62.9)		
Parity							
1–3 children	104(69.3)	15(46.9)	.015*	102(69.4)	17(48.6)	119(65.4)	.029*f
4 or more	46(30.7)	17(53.1)		45(30.6)	18(51.4)	63(34.6)	
Mode of delivery							
Normal	120(80.0)	31(96.9)	.019f*	116(78.9)	35(100.0)	151(83.0)	.001f*
C-section	30(20.0)	1(3.1)		31(21.1)	0(0.0)	31(17.0)	
Received family support							
Yes	79(53.0)	3(33.3)	.314f	74(54.4)	8(36.4)	82(51.9)	.167f
No	70(47.0)	6(66.7)		62(45.6)	14(63.6)	76(48.1)	
Received partner/friend support							
Yes	116(77.3)	6(18.8)	.000*	111(75.5)	11(31.4)	122(67.0)	.000f*
No	34(22.7)	26(81.3)		36(24.4)	24(68.6)		
Distance to health facility (KM)							
< 5 KM	56(37.3)	19(59.4)	.029f*	52(35.4)	23(65.7)	75(41.2)	.001f*
> 5 KM	94(62.7)	13(40.6)		95(64.6)	12(34.3)	107(58.8)	
Friendly health workers							
Yes	87(59.6)	8(34.8)	.040f*	85(58.2)	10(43.5)	95(56.2)	.136f
No	59(40.4)	15(65.2)		61(41.8)	13(56.5)	74(43.8)	
Healthcare workers available							
Yes	99(66.9)	4(21.1)	.000*	93(63.7)	10(47.6)	103(61.7)	.229f
No	49(33.1)	15(78.9)		53(36.3)	11(52.4)	64(38.3)	

* p-value (p<0.05, chi-square test)

Abbreviation: KM Kilometre, f-fisher exact test

### Poor ANC attendance during pregnancy

Eighty-two percent of the women living with disabilities reported that they had attended ANC, and more than half had delivered their babies with the aid of skilled birth attendants at different health facilities. Contrary, midwives reported that few women with disabilities attended ANC.

*“We receive at least one disabled pregnant woman in a month or after three months, women with a disability rarely come to the health facility in Busiro HSD. They may be going to Entebbe Grade B hospital which is far and costly.”* (KI Midwife, Kajjansi HC IV)

Key informants mentioned that a few disabled women visit Kajjansi HC for ANC and delivery service since most preferred hospitals are better equipped. Accordingly, few pregnant women living with disabilities visit health facilities in Busiro HSD as illustrated in the excerpts below;

*“NO, only a few pregnant disabled women attend ANC for screening. After identification, midwives refer the disabled pregnant women to Entebbe hospital for future management. This is because there are no theater services and few interventions have been put in place to cater for the needs of women with disabilities at Kajjansi H/C 1V in Busiro South HSD, Wakiso district,”* KI, Kajjansi HC IV.

*“Also, Kasanje Health H/C III is the only government-run health facility in Kasanje town council serving seven parishes and more than 30 villages. We do not have a specialized maternity unit to cater for disabled women.”* (KI, Kasanje HC III)

### Myths and misconceptions on maternal health-seeking behaviour among disabled women

#### Use of family planning

One of the misconceptions of physical disability is that the communities believe that women with disabilities got it from their mothers who used family planning while pregnant.

*'Some community members say that my sister became lame because my mother used family planning when she was pregnant and this makes my mother feel bad.”* (FGD 3 of relatives of women with disability)

#### Used Herbs pregnant

Most community members blame disability with parents saying that their mothers took herbal medication while pregnant. This has discouraged many women with disabilities feel out of place and makes some of them shy away from going to health facilities while pregnant. In addition, some communities do not respect women with disabilities at all for they believe that they have no economic value and are more of a burden as illustrated.

*“People think that women with disabilities are useless, as a result, they hate us. Victimization and social stigma have made us lack the confidence to access and use maternal health services. We prefer to go to the traditional birth attendants because they treat us with respect and care about our needs. And the fear of stigma hinders us from delivering our babies at the hospital.”* (FGD 3 with CHWs)

### Cultural beliefs

Participants of the FGDs believed that women with disabilities were born prematurely and are likely to have disabled children. They argued that these types of women couldn't have children normally because their bones are rigid, which has hindered them from utilizing hospitals fearing to be operated upon and are laughed at even when they give birth to normal children.

*“Some people say how can a disabled woman give birth to a normal child? One of the relatives of the woman with a disability complained*. (*FGD 1* with relatives of women with disability)

### Maternal Health Care specialist in Busiro HSD

A few disabled women visit health care services in Busiro HSD because there are no specialized maternal and child care units. Accordingly, they are inadequately trained to deliver disabled women who may have birth complications as illustrated in the excerpts below.

*“We have not received special training in handling disabled pregnant women at the respective health facilities. And health care workers attending to mothers with disabilities, normally refer them to other health facilities although they deliver some during emergencies.”* (KI, Kasanje HC III)

*“When identified early during ANC, pregnant women with disability are advised to visit better-equipped public health facilities with more specialized services for better management. However, if the disabled woman comes in when already in labor, we try to manage using the available resources.”* (KI, Kasanje HC III)

*“During ANC screening, the midwives assess if the woman with a disability will be able to have a baby naturally (normal delivery or not) to avoid complications when they are referred to Entebbe Hospital. There is no theatre, so timely referral for difficult cases in done”* (KI, Kajjansi HC IV)

### Maternal health-seeking behavior of women disability in Busiro HSD

#### ANC attendance

Maternal health-seeking behavior at ANC was more likely when mothers were below 30 years of age (OR 0.325, 95%CI 0.137-.768, p0.010) than when they had less than 3 pregnancies (OR 0.295, 95%CI: 0.130–0.667, p0.003) and when a mother had less than three children (OR 0.390, 95%CI 0.180–0.848, p0.018). Equally, maternal health-seeking behavior of a disabled woman was more likely if she perceived a normal delivery (OR 7.750, 95% CI 1.017–59.075, p0.048), distance to the health facility (OR 2.453, 95%CI 1.126–5.347, p0.024). The friendliness of the health provider towards a woman with a disability and their availability at the health facilities was positively associated with maternal health-seeking behavior (OR .362, 95% CI: .144-.907, p0.030). Conversely, maternal health-seeking behavior was not associated with the marital status of a woman living with a disability (Table 2).

**Table 2:** A summary of factors associated with maternal health-seeking behaviour of disabled women

		Binary Logistic Regression			
		
		ANC attendance		Place of delivery	
**Characteristics**	**Level**	**OR**	**95% CI**	**OR**	**95% CI**
Maternal age (years)	18–30	.325*	(.137, .768)	.332*	(.146,758)
	> 30	Ref		Ref.	
Marital status	Single	.464	(.082, 2.619)	.299	(.044, 2.008)
	Married	.831	(.209, 3.302)	.831	(.209, 3.302)
	Separated	1.444	(.351, 5.947)	2.167	(.539, 8.711)
	Widowed	Ref		Ref.	
Number of pregnancies parities	1–3	.295*	(.130, .667)	.396*	(.185, .848)
	≥ 4	Ref.		Ref.	
	1–3 children	.390*	(.180, .848)	.417*	(.197, .882)
	≥ 4	Ref.		Ref.	
Method of delivery	Normal	7.750*	(1.017, 59.075)		
	C-section	Ref.			
Received family support	Yes	.443	(.107, 1.838)	.479	(.189, 1.216)
	No	Ref.		Ref.	
Received partner/friend support	Yes	.068*	(.026, .178)	.149*	(.066, .333)
	No	Ref.		Ref.	
Distance to health facility (KM)	< 5	2.453*	(1.126, 5.347)	3.502*	(1.612, 7.605)
	≥ 5	Ref.		Ref.	
Friendly health workers	Yes	.362*	(.144, .907)	.552	(.227, 1.3341)
	No	Ref.		Ref.	
Healthcare workers available	Yes	.132*	(.042, .419)	.518	(.206, 1.301)
	No	Ref.			

Note: 95% confidence interval for odds ratios (OR) are in brackets; *p<0.05 at 5% level of significance, Ref. Reference category

#### Place of delivery

The results above show that the odds of a woman (>30 years) to disability delivering her baby at a health facility were .33 times likely than their counterparts that were below 30 years of age (OR .332, 95% CI.146-.758, p0.009). Conversely, the place of delivery was not associated with the marital status of a woman living with a disability. A disabled woman with less than 3 pregnancies delivering her baby at a health facility was 39 times that of a disabled woman with more than three children (OR 0.396, 95%CI: 0.185–0.848, p0.017). The mode of baby delivery was not associated with health facility delivery. Equally, the odds of a disabled woman delivering their baby at the health center if they were supported by their friends was14 times that of disabled women who had not received support (OR 0.149, 95%CI 0.066–0.333, p<0.001). A disabled woman was three times likely to deliver at the health facility if she lived less than 5KM (OR 3.502, 95%CI 1.612–7.605, p<0.002). The odds of a disabled woman with less than three children delivering her baby at the health facility was .41 times that of a woman with more than three children (OR .417, 95% CI: .197-.882, p0.022). A disabled woman delivering her baby at health was associated with the number of previous pregnancies (OR .396, 95% CI: 0.185-.848, p0.017).

## Discussion

In our study, the overall ANC attendance among women with disabilities in Busiro South HSD was high (82.3%). A previous study by Omona demonstrated a similar finding in that more women with disabilities attended ANC and delivered their babies at government-run health facilities^15^. As the ANC attendance was less than 100%, it is plausible that barriers related to healthcare seeking could have hindered their ANC attendance. Most maternal health facilities are inaccessible for women with mobility disabilities and few that are, providers do not have the needed equipment like adjustable examination beds and other specialized services for disabled pregnant women. This is affirmed by 43.8% of the disabled women who reportedly had no means of transport to visit the public health facility. Furthermore, 58.8% of the disabled women resided more than 5kilometre from the health facility. As one of the socio-demographic factors that showed a significant association, distance to the health facility is a determinant of healthcare-seeking. This concurs with the studies that were conducted by Ledger in UK^16^ and Ganle^5^ in Ghana where the long-distance women with a disability had to travel with their caretakers made it difficult for them to access maternal health care especially those with visual impairments. This means that a bigger number of women with disabilities stay far away from the health facilities, thus there is a need to take health care services to the individual communities to increase utilization. There is need to ensure physical accessibility of maternal and child care services for women with disabilities^2^. Alternatively, the provision of financial support from friends of partners as one of the socio-demographic determinants of healthcare-seeking is plausible. This would support their healthcare access means and utilities while attending the ANC. Similar to earlier reports, financial support fostered the long distance to be covered by pregnant women who lived further from health facilities and provided them with relief to obtain most of the hospital needs^13^,^15^. Due to such barriers, it emerged from the qualitative findings that women with disabilities opted to deliver from nearby service providers including TBAs and private maternity clinics. Consistent with previous reports^9^,^13^,^15^, the observed trend necessitates the need to overcome such barriers and raise awareness about the importance of skilled labor-aided hospital delivery among women with disabilities.

This study found cultural misconceptions as some communities blamed disability on parents saying that their mothers took herbal medication while pregnant. There is a need to provide exclusive informational resources tailored to the need of women with disabilities on pregnancy like books, magazines, posters, brochures, and community outreaches^17^. In addition, some communities did not respect women with disabilities at all because they believe that they did not contribute significantly to the development of their community. Women with disabilities encounter negative attitudes about pregnancy and parenting from their family members, health providers, and community members^18^. This deters pregnant women with disabilities from accessing and utilizing maternal health services increasing pregnancy health risks. Furthermore, some participants believed that women with disabilities were likely to undergo caesarean section while giving birth. They argued that these types of women couldn't have children normally because their bones are rigid; consequently, this hindered them from utilizing hospitals fearing to be operated on at subsequent delivery. The physical disability affects the pelvic bones, so there is a need for specialized care in handling pregnant women with a disability and adequate assessment before baby delivery to avoid complications that may arise during childbirth. However, healthcare providers are not often trained to provide reproductive healthcare to women with disabilities^18^ and where health workers have specialized training, they are not located near all women who need their needs. Although not well researched, misconceptions related to physical disability have been reported to largely portend healthcare seeking 9, 12, 13, 19, 20. This complex phenomenon may foster avoidable pregnancy complications and aggravate death. This requires a concerted approach to elude falsehoods and promote healthcare seeking.

The maternal age, number of pregnancies, parity, friendliness of health care providers, and availability of health providers showed a statistically significant association with maternal health-seeking behaviour of women living with disabilities in Busiro HSD, Wakiso district. Consistent with previous reports, women who had delivered more than three children had fewer deliveries in health facilities than those that had delivered more than three children. This is in agreement with the study conducted by Kifle in Ethiopia and Kawungezi in Uganda^20^ that showed a decrease in seeking maternal services with increased parity^19^. However, the findings are in contrast with a study done in Malawi which indicated that high parity women sought maternal services more such as ANC services compared to their counterparts 21. Premised on this, there is a need to stress the importance of hospital-managed delivery on maternal and child health outcomes. One such way is ensuring the availability of health providers. There is a need to collect routine data about pregnancy among women with disabilities to understand local issues, establish trends and priorities for this population. This would enable the government to provide better maternal healthcare services for women with disabilities.

Our study established the need for maternal health child specialists as few disabled women visit health care services due to the shortage of specialized maternal and child care units. Accordingly, they are inadequately trained to deliver disabled women who may have birth complications. This is achievable through policy frameworks that ensure sufficient and well-motivated midwives. This corroborates well with previous studies that were carried out in Uganda and Zimbabwe indicating that people with disabilities were still treated as any other normal person 22, 23 forgetting that they have special care needs. In addition, the friendliness of health care providers was found to influence health-seeking behavior among women with disabilities in our study. Previous studies in Uganda indicated that the utilization of maternal health care by a woman with a disability is affected by the poor attitude of health workers^14^, lack of clear direction on services offered at the different facilities, crowding, and lack of privacy^20^,^23^,^24^. The friendliness of health care providers is critical since previous studies in Uganda by Ahumuza and Mulumba further indicated that these women with disabilities were despised and mistreated by health workers^13^. This concurs with the study findings by Ledger that found that most health care workers were not trained to be cautious and polite with their patients^21^,^25^,^26^. Uganda needs that maternal healthcare services are situated to provide holistic, accessible care to women with disabilities before, during, and after their pregnancies. Although many healthcare facilities in Busiro HSD offer antenatal care, eMTCT services, home visits, family planning and immunization that are critical for women transitioning through pregnancy and early motherhood^12^. There is a need to include women with disabilities in such services to reduce maternal health disparities experienced by this population and improve their pregnancy outcomes. Thus, the ministry of health should develop new programs for pregnant women and new mothers with disabilities.

## Limitations of the study

The results of this study ought to be interpreted in light of the shortcoming that: a) the respondents gave self-reported information that cannot be ascertained for accuracy, 2) recall bias. The study had a small sample size because only found 182 women with disabilities that had consented had given birth twenty-four months and many confounding variables may have influenced the results.

## Conclusion

The majority of disabled women attended ANC during pregnancy and had delivered their babies with the aid of skilled birth attendants at different health facilities. However, misconceptions and cultural beliefs about the physical disability state and pregnancy affected their healthcare-seeking. Numerous factors of maternal age, number of pregnancies, children ever born, mode of delivery, receiving financial support from friends of partners, distance to the health facility, friendliness of health care providers, and availability of health providers were statistically associated with health-seeking among women living with a disability. Therefore, there is a need to raise awareness on pregnancy management among the disabled through community outreach and routine checkup. In addition, a multi-sectoral approach is needed to encourage women with disabilities to use health services through the provision of user-friendly maternal facilitates, and regular training of health workers in handling women with disabilities to attract more of them to the health facilities.

## List of abbreviations

HSD: health sub-district
ANC: Antenatal care

## Declarations

### Availability of data and materials

We did not obtain consent to share the data obtained from the questionnaire, however, the datasets used may be availed on request from the corresponding principal investigator.
